# Quality of life and mental health status in caregivers of pediatric patients with nephropathic cystinosis

**DOI:** 10.1186/s13023-024-03417-1

**Published:** 2024-11-05

**Authors:** Karina González, Teresa Eixarch, Laura Nuñez, Gema Ariceta

**Affiliations:** 1 Service of Pediatric Nephrology, Vall d’Hebron University Hospital, Barcelona, Spain; 2 Departments of Pediatrics, Obstetrics and Gynecology, and Preventive Medicine, Universitat Autonoma de Barcelona, Barcelona, Spain

**Keywords:** Nephropathic cystinosis, Health-related quality of life, Caregivers, Anxiety, Depression

## Abstract

There are few studies assessing psychological burden and quality of life (QoL) in caregivers of pediatric patients with nephropathic cystinosis, a severe chronic disease. This observational, single-center study aimed to explore the levels of anxiety, depression, care burden, and QoL status in caregivers of patients with nephropathic cystinosis. The Hospital Anxiety and Depression Scale (HADS), the Zarit Caregiver Burden Scale, and the Short Form-36 (SF-36) were administered to caregivers of pediatric patients with nephropathic cystinosis. Nine caregivers of pediatric patients with nephropathic cystinosis participated in the study (6 boys and 3 girls; mean age, 12.6 ± 4.2 years). All participating caregivers were the patient’s mothers. Of the 9 caregivers, 6 showed anxiety/depression and 4 severe care burden. Overall, SF-36 QoL domains with a worse perception by caregivers were ‘general health’ and ‘health change over time’. Mothers without depression/anxiety and low care burden had better QoL perception (*p* = 0.02). All caregivers with high care burden showed anxiety/depression. In our study cohort, caregivers of pediatric patients with nephropathic cystinosis showed high levels of anxiety/depression, high care burden, and impaired QoL, highlighting the importance of detecting psycho-social issues to implement strategies that relieve family stress and improve coping strategies.

## Introduction

Nephropathic cystinosis (NC) is an ultra-rare autosomal recessive genetic-metabolic disorder with an estimated incidence of 1:100,000-200,000 live births. Lack of function mutations in the *CTNS* gene encoding the cystinosin, a lysosomal membrane transport protein, causes the pathological accumulation of cystine crystals within the lysosomes [1]. This leads to cell death, which manifests characteristically with early kidney involvement and later progressive multisystemic deterioration over time [2]. After clinical suspicion, the diagnosis is confirmed by quantifying white blood cell (WBC) cystine levels above 1 nmol hemicystine/mg protein in untreated patients, together with genetic identification of the pathogenic variants in the *CTNS* gene [1].

In NC, newborns and infants younger than 6 months are usually asymptomatic or show a slight growth impartment. However, within 6 and 12 months old months, the disease manifests as a severe renal Fanconi syndrome characterized mainly by polyuria, polydipsia, vomiting, and failure to thrive. Over time, the disease progresses to chronic kidney disease (CKD) and kidney failure by the age of 10–12 years if untreated, requiring renal substitutive therapy (dialysis and transplant afterward) [1].

Besides kidney involvement, the natural history of the NC in the absence of specific treatment is also characterized by progressive extra-renal complications that develop as patients age, including corneal crystal deposition causing photophobia and visual impairment, hypothyroidism, hypogonadism, diabetes mellitus, gastrointestinal problems, bone disease, muscular weakness and wasting, and respiratory and central nervous system manifestations [1]. Nowadays, the course of the disease has dramatically changed with the availability of kidney replacement therapy and specific treatment with cystine depletion therapy (CDT), which led to a significant increase in life expectancy, prevented progression, delayed the need for kidney transplantation, and reduced extra-renal complications [2].

CDT, which comprises oral and ocular cysteamine, decreases the WBC cystine content and represents the mainstay of treatment in patients with NC. Cysteamine therapy should be initiated immediately after diagnosis of the disease and continued throughout life. It helps to reduce WBC cystine levels, which improves growth, preserves renal and extra-renal organ function, and increases life expectancy [3, 4].

Unfortunately, cysteamine causes significant side effects, such as gastrointestinal symptoms, halitosis, and unpleasant body odor. Furthermore, treatment adherence is often compromised by the burdensome and strict lifelong dosing schedule, even at night in case of immediate-release cysteamine, leading to chronic sleep deprivation for patients and caregivers, especially those who take care of infants and children. All these factors significantly impair the quality of life (QoL) of patients and families, negatively impact patient compliance, and may jeopardize clinical outcomes [5–7]. Therefore, disease complications and side effects of cysteamine frequently impact relationships, autonomy, and social life [4].

There is growing attention to the burden and psychosocial stress experienced by the caregivers of children with CKD [8] and its impact on patient outcome [9]. Cystinosis also causes a severe impact on the family, who needs to adapt to the complexity of caring for a patient with a chronic, rare disease and a very demanding treatment regimen. Detecting and treating mental health issues specifically in patients with cystinosis and their caregivers, focused on the disease singularities, is an unmet need; it might help relieve their stress and burden, increase adherence, and empower the patient [10].

A recently published review article on Health-related quality of life in cystinosis only detected 5 articles describing the QoL of NC patients within all the literature. Despite the lack of qualitative research on this topic and the need to develop a cystinosis-specific QoL patient-reported outcome measurement (PROM), the authors concluded that QoL was reduced in cystinosis patients compared to healthy individuals. Nevertheless, no studies have been performed to evaluate the effect of this devastating disease on the caregiver’s physical and mental health [11]. Thus, this research aimed to explore the impact on the caregivers of pediatric NC patients cared at our institution, by assessing their anxiety, depression, care burden, and QoL.

## Methods

This observational, single-center study was conducted at the University Hospital Vall d’Hebron (Barcelona, Spain). There were no inclusion/exclusion criteria. The Ethics Committee of the HVH approved the study. Informed consent was obtained from all participants. This study aimed to assess the levels of anxiety, depression, care burden, and QoL status in caregivers of patients with NC. We also compared the QoL status based on the presence/absence of anxiety/depression or care burden level. To this end, the Hospital Anxiety and Depression Scale (HADS), the Zarit Caregiver Burden Scale, and the Short Form-36 (SF-36) were used.

The HADS test was used to evaluate the anxiety/depression status of caregivers. This questionnaire is a self-assessment tool to detect depression and anxiety states, divided into different subscales. The scale comprises 14 items, which can be graded on a 0–3 scale, with total scores ranging from 0 to 21 and higher scores representing more severe depression or anxiety. For each subscale, scores < 7 are negative for anxiety or depression, 8–10 represent borderline anxiety or depression, and ≥ 11 indicate probable anxiety or depression [12]. In this study, HADS scores were classified into absence (< 11) or presence (≥ 11) of anxiety/depression.

The Zarit Caregiver Burden Scale is a 22-item self-report measure of caregiver burden. Each item can be scored from 1 (‘none’) to 5 (‘nearly always’), with a total score from 22 to 110 (most severe burden). Total scores can be divided into 3 levels: no burden (22–46 points), mild burden (47–55) and severe burden (56–110). To simplify, we classified Zarit scores into 2 groups: low burden (< 55) and high burden (≥ 55). The Zarit Caregiver Burden Scale, an adapted and validated version in Spanish, was the one used in this study [13].

The SF-36 is a generic instrument to measure health-related QoL. This instrument comprises 36 questions organized across 8 dimensions: physical functioning, bodily pain, social functioning, mental health, role limitations from emotional health problems, vitality, general health, and health change over time. Total scores range from 0 to 100, with 100 reflecting the best QoL status. The validated Spanish version was used in this study [14].

The questionnaires were administered once at the time of inclusion. The data collected from the caregivers were the relationship with the patient (mother/father) and the employment status at the time of the inclusion. Data from patients, such as gender, age at inclusion, kidney disease status, renal transplant, treatment, and extra-renal complications, were also recorded.

## Results

A total of 22 caregivers (mothers and fathers) of patients with NC currently managed by the Pediatric Nephrology Department of Vall d’Hebron Hospital were invited to participate in the study, and 9 of them accepted. They were all mothers of 6 boys and 3 girls (Table 1). Four patients had CKD stage 1, 1 had CKD stage 2, and 4 had CKD stage 3. Four patients had undergone a kidney transplantation (44%). The mean age of the patients was 12.6 (± 4.2) years.

**Table 1:** Demographic and clinical characteristics of caregivers and patients at the time of the study

Caregivers			Patients							
Caregiver Number	Relationship	Working (Y/*N*)	Sex	Age at diagnosis (years)	Current age (years)	CKD stage	Kidney transplant	Cysteamine treatment	Extrarenal manifestations	Concomitant pathologies
1	Mother	no	Girl	1	19	3	no	IR cysteamine	no	no
2	Mother	no	Boy	2	17	1	yes	IR cysteamine	no	no
3	Mother	yes	Girl	1	16	3	no	IR cysteamine	Endocranial hypertension, shunt, **severe** impaired vision	no
4	Mother	yes	Boy	1	13	2	no	IR cysteamine	no	no
5	Mother	no	Boy	1	12	1	no	ER cysteamine	no	no
6	Mother	no	Girl	0.8	6	1	no	IR cysteamine	no	gastrostomy
7	Mother	no	Boy	1	12	3	yes	IR cysteamine	Endocranial hypertension, shunt	no
8	Mother	yes	Boy	1	13	3	yes	IR cysteamine	Hypothyroidism	no
9	Mother	yes	Boy	1.2	6	1	no	IR cysteamine	no	no

Abbreviations: CKD, chronic kidney disease; ER, extended-release; IR, immediate-release

**Fig. 1:**
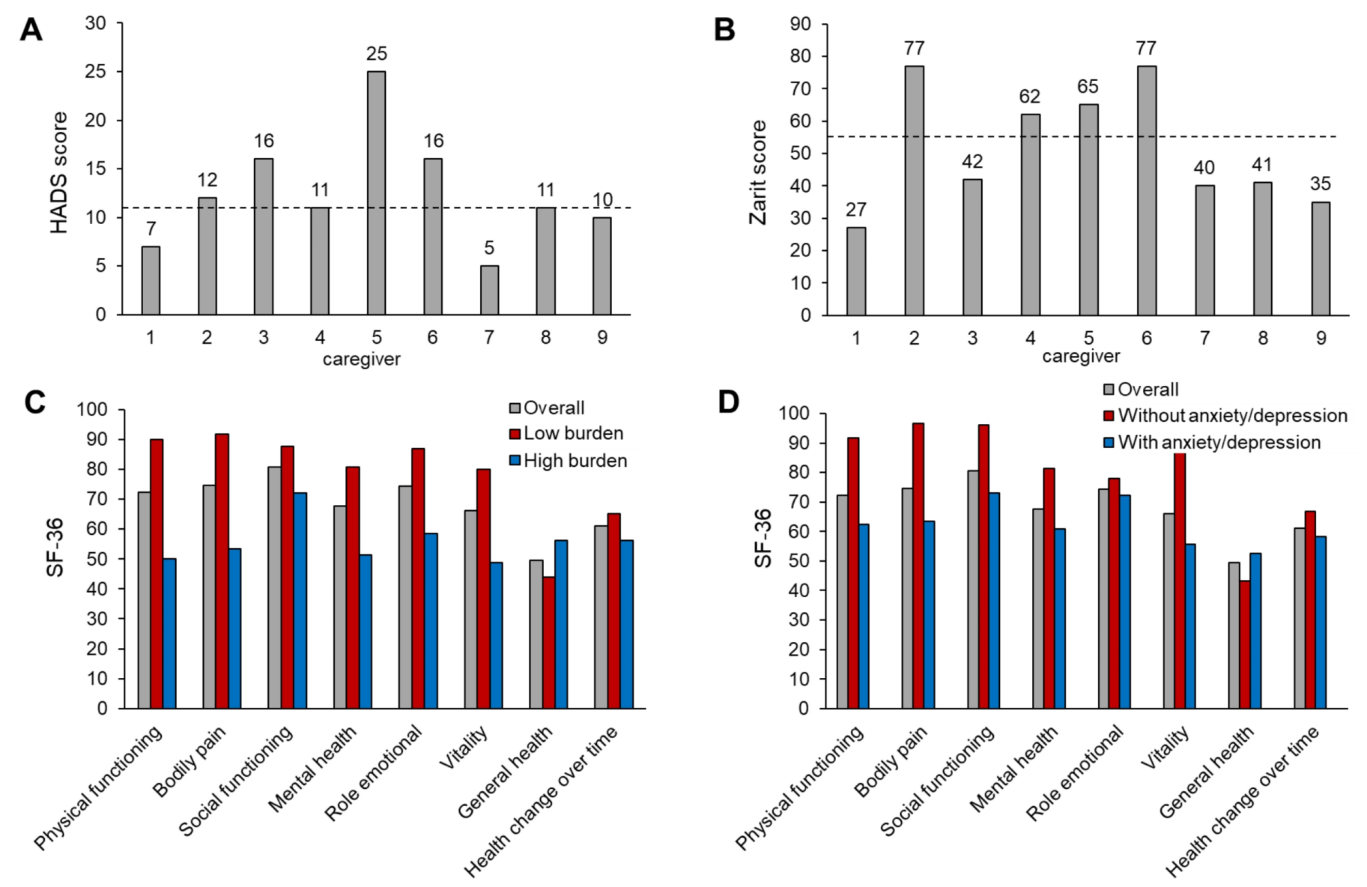
Levels of anxiety/depression, care burden and quality of life status in caregivers of patients with nephropathic cystinosis. Bar graphs show the Hospital Anxiety and Depression Scale (HADS) **(A)** and Zarit Caregiver Burden score **(B)** for each caregiver. Mean Short Form-36 (SF-36) scores in the overall caregiver cohort are shown based on the burden level **(C)** or the presence/absence of anxiety/depression **(D)**. A HADS score of > 11 indicates the absence of anxiety/depression, and ≤ 11 indicates its presence. For Zarit scores, > 55 indicates low burden, and < 55 indicates high burden (dashed lines)

Six out of 9 caregivers showed a HADS score above 11 (mean, 12.5), indicating that 66.7% of the caregivers suffered from anxiety/depression. Regarding the care burden, the mean Zarit score was 51.7. In this questionnaire, five caregivers showed low burden (score < 55), but five of them reported high-severe burden (score ≥ 55) (44%) (Fig. 1). The 4 caregivers who reported high care burden also suffered from anxiety/depression (Fig. 1, caregivers 2, 4, 5 and 6).

Overall, the SF-36 scores were between 40 and 85 in all domains, indicating a medium-low QoL in caregivers. The domains with a worse perception by caregivers were ‘general health’ and ‘health change over time’ in the entire study population. QoL perception was better in caregivers with a low burden than caregivers with a high burden, except for the ‘general health’ subdomain. The most remarkable differences in QoL perception between caregivers with low or high burdens were observed for the ‘physical functioning,’ ‘bodily pain’, and ‘vitality’ domains (Fig. 1).

## Discussion

Caring a child with chronic and complex disease can be extremely challenging with consequences on caregivers’ mental health [11, 15]. NC is an ultrarare treatable condition that manifests in early infancy and requires live-long treatment and often parental support [1–3]. However, very little research has focused on caregivers’ mental health and QoL in NC. This study aimed to assess the presence of anxiety-depression, care burden and impact of QoL in caregivers of pediatric patients with NC, measured by the HADS, the Zarit Caregiver Burden Scale and SF-36 test. Based on the literature, we presumed finding a direct correlation between patient CKD severity, NC progression and lack of access to ER-cysteamine, among other factors with increasing caregiver mental health issues and reduced QoL [11, 16–18].

Indeed, in this study, we observed that 6 out of 9 caregivers suffered anxiety/depression (66.7%), and 4 (44.4%) showed severe care burden. Further, the study population presented decreased QoL perception, mostly in the ‘general health’ and ‘health change over time’ domains. Thus, caregivers # 7 and 8 suffered from anxiety/depression, high burden and reduced QoL possibly related to significative CKD in a previously kidney transplanted sibling [18]. Caregiver #3 also scored for high burden of care that may be attributed to caring a visual disable teenager suffering from a severe central nervous system adverse event of NC [1, 3].

Families of children with NC face new challenges [19] related to chronic medication regimen, treatment-related side effects, frequent medical appointments and hospitalizations, and the need to cope with complex situations that involve different spheres of life [16]. The impact of the disease on the families’ lives is also justified by parent’s concerns related to the loss of kidney function, growth, and digestive problems [10]. Despite we cannot confirm that presence of anxiety-depression and reduced QoL in this study caregivers are directly associated with caring for a child with cystinosis, given the small study sample, similar experiences have been reported in other rare diseases, and different authors observed a relation between disease care burden and caregivers’ mental health issues [10].

In a prior qualitative study reporting the experiences of patients with cystinosis and their parents, participants identified family dedication as a relevant contributor to the survival of individuals with cystinosis [20], and often limited availability of disease information and access to specialists with expertise in cystinosis, given the rare nature of the disease [21]. Moreover, children and their families may feel burdened by difficulties in managing patient needs, the strict dosing schedule to treat cystinosis, and the associated side effects such as gastrointestinal symptoms or unpleasant body and breath odor [15].

However, in our study, there was not a clear relationship between maternal anxiety/depression, disease burden and cystinosis progression in every case. Other factors such as personal abilities, and social factors, could affect these observations [22], highlighting the complexity of the topic. Therefore, caregiver #9 exhibited anxiety-depression and care burden with a well-controlled child, and caregiver #5 should had scored the best for mental health considering her child had a good clinical condition and less treatment burden using ER cysteamine. Those findings may reflect other key aspects which are not addressed in the current analysis and represent a limitation of our study. Thus, it is important to consider other features such as families socioeconomic background, social support, unemployment, and geographic distance to the expert center that are known to impact pediatric CKD and NC outcome. Finally, caregiver personal psychosocial stressors, resilience tools, feelings of isolation from peers [16] and lack of support of specific aspects of families with rare diseases, such as the need to stop working to care for the child [19], have shown a closer association with families issues than to the severity of the disease itself [11, 17, 22, 23].

Further, transition to adult care and continuum of care of vulnerable population of children with NC is known to represent one of the major caregivers’ concerns [10, 11, 20, 24]. Thus we could hypothesized that anxiety-depression with negative impact on QoL observed in caregiver #1 may be attributed to transition and emerging psychologic issues in young adults with NC during transition [25]. Similar studies in young adult patients with cystinosis were performed recently, demonstrating that the disease can affect all QoL domains in these populations [2, 26, 27]. Some differences were found between adults and pediatric patients; adults were more concerned about the cystinosis complications and the side effects of cysteamine, affecting relationships, autonomy, and social life, whereas impairment of QoL in children was more related to school attendance and social life [20, 28].

Based on our results, we pose the need of conducting research in parents of pediatric patients with NC to build further knowledge and evidence about the potential impact on parental mental health and QoL, in order to focus on the psychological impact on the parent and set up coping and support strategies if applicable.

The main limitations of the study were the small sample size, absence of socioeconomic and family background data, and the lack of psychological follow-up of those caregivers. Further, the study was conducted in a single center, which limited the data collection and analysis.

In our cohort, we observed that more than half caregivers of pediatric patients with NC suffered anxiety, depression, care burden, and impaired QoL, that may impact disease outcome and family well-being. However, we did not observe a direct association between maternal mental health problems and disease progression, with highlights the need to further consider other individual, socioeconomic and health care features role on mental health. This small study aim was to provide new evidence of mental health and QoL impact on caregivers of pediatric NC patients. It can represent a starting point to set up further exploratory studies in larger multicenter cohorts to improve further knowledge in caregivers’ psycho-social issues to initiate strategies that mitigate family stress and facilitate patient empowerment, adherence, and emotional health.

## Data Availability

The authors confirm that the data supporting the findings of this study are available within the article.

## Abbreviations

CKD: Chronic kidney disease
HADS: Hospital Anxiety and Depression Scale
QoL: Quality of life
SF-36: Short Form-36
